# The Impact of Sprint Interval Training Frequency on Blood Glucose Control and Physical Function of Older Adults

**DOI:** 10.3390/ijerph17020454

**Published:** 2020-01-10

**Authors:** Simon Adamson, Mykolas Kavaliauskas, Ross Lorimer, John Babraj

**Affiliations:** 1 Division of Sport and Exercise Sciences, Abertay University, Dundee DD1 1HG, UK; s.z.adamson@dundee.ac.uk (S.A.); ross.lorimer@abertay.ac.uk (R.L.); 2 School of Applied Sciences, Sport, Exercise and Health Sciences, Edinburgh Napier University, Edinburgh EH11 4DY, UK; M.Kavaliauskas@napier.ac.uk

**Keywords:** ageing, sprint interval training, physical function, blood glucose control

## Abstract

Exercise is a powerful tool for improving health in older adults, but the minimum frequency required is not known. This study sought to determine the effect of training frequency of sprint interval training (SIT) on health and physical function in older adults. Thirty-four (13 males and 21 females) older adults (age 65 ± 4 years) were recruited. Participants were allocated to a control group (CON *n* = 12) or a once- (*n* = 11) or twice- (*n* = 11) weekly sprint interval training (SIT) groups. The control group maintained daily activities; the SIT groups performed 8 weeks of once- or twice-weekly training sessions consisting of 6 s sprints. Metabolic health (oral glucose tolerance test), aerobic capacity (walk test) and physical function (get up and go test, sit to stand test) were determined before and after training. Following training, there were significant improvements in blood glucose control, physical function and aerobic capacity in both training groups compared to control, with changes larger than the smallest worthwhile change. There was a small to moderate effect for blood glucose (*d* = 0.43–0.80) and physical function (*d* = 0.43–0.69) and a trivial effect for aerobic capacity (*d* = 0.01) between the two training frequencies. Once a week training SIT is sufficient to produce health benefits. Therefore, the minimum time and frequency of exercise required is much lower than currently recommended.

## 1. Introduction

Ageing is characterised by a gradual reduction in skeletal muscle mass of approximately 30%–50% between the ages of 40 and 80 years old [1]. The loss of skeletal muscle mass results in muscular weakness, which makes carrying out everyday tasks more difficult and increases the risk of physical disability with age [2]. This loss of skeletal muscle mass is associated with a reduction of mitochondrial content, leading to a loss of aerobic capacity with age [2]. The loss of aerobic capacity is also associated with a decline in physical function in later life [3]. As well as changes in aerobic metabolism, there is a decline in blood glucose control with ageing [2]. Insulin resistance with ageing has been associated with increased adiposity, impaired beta cell response and impaired muscular response to the rise in circulating glucose [4]. Therefore, maintaining muscular function, metabolic health and aerobic fitness is extremely important for ensuring healthy outcomes in later life.

Aerobic and resistance training have been shown to have positive effects on the ageing processes, attenuating declines in muscle mass, physical function and metabolic health [5]. Following 16 weeks of aerobic exercise (three progressing to four sessions per week at 70% VO_2_ max), there was a 9% increase in VO_2_ max which was the same regardless of age [6]. Likewise, twenty weeks of aerobic exercise (four sessions per week at 70% VO_2_ max) have also been shown to improve VO_2_ peak by 8% and sit to stand performance by 9% [7]. Similar improvements in sit to stand (10%) have also been seen with twenty weeks of resistance training (three sessions per week at 70% 1 repetition maximum, (RM) [7]) whilst 8 weeks of resistance training (three sessions per week at 75%–80% 1 RM) resulted in mitochondrial adaptations with a 20–40% increase in expression of key enzymes related to aerobic metabolism in skeletal muscle [8]. This increase in aerobic metabolism of the skeletal muscle was associated with a lower cardiorespiratory response to submaximal exercise [8]. In contrast, the response of blood glucose control has been shown to be blunted in older adults (5% improvement in insulin sensitivity) compared to younger adults (72% improvement in insulin sensitivity) following 16 weeks of aerobic exercise. After 6 months of aerobic exercise (three times per week at 70% VO_2_ max), the area under the blood glucose curve was reduced by 3.1% and 2 h post blood glucose concentration was reduced by 3.6% [9]. When weight loss was included with aerobic exercise then the response was much greater (17.3% reduction in area under the curve, 22.5% reduction in 2 h blood glucose; [9]). Following 8 weeks of resistance training, blood glucose control is improved, with a 20% reduction in the area under the blood glucose curve following ingestion of 75g carbohydrate and lower 2 h blood glucose concentration [8]. The greater adaptation of blood glucose control seems to be related to the ratio of muscle mass to intramuscular fat mass with both aerobic and resistance exercise. Despite this, participation in traditional training modalities is low in older adults [10], with low adherence after supervised training [11]. Therefore, there is a need to identify different approaches to exercise with older adults.

Sprint interval training (SIT) can be defined as repeated supramaximal intervals of a duration of less than 30 s, with a longer period of recovery [12]. SIT has been shown to improve blood glucose control in younger adults when performed 3 times per week (4–6 30 s sprints with 4 min recovery, [13]). Likewise, twice-weekly SIT (10 × 6 s sprints with 60 s recovery) has been shown to improve blood glucose control (6% reduction in glucose area under the curve, 11% reduction in 2 h glucose concentration) and physical function (20% decrease in timed get up and go) in middle aged people [14]. In older adults, twice-weekly SIT (10 × 6 s sprints with 60 s recovery) has been shown to improve aerobic capacity (8% increase in predicted VO_2_ max) and physical function (11% decrease in timed get up and go) after 6 weeks [15]. Following 10 weeks of the same SIT protocol, physical function was shown to be improved along with arterial stiffness and circulating lipids [16]. The lowest frequency reported for SIT is one session every 5 days (6 × 30 s sprints at 50% peak power with 3 min recovery) which resulted in a clinically relevant increase in peak power and greater lean body mass in older adults after 6 weeks [17]. This suggests that less than twice per week may be a sufficient training stimulus for improvements following SIT. 

Despite the effectiveness of SIT in promoting health benefits, it is not clear what the minimum frequency of training that is required to deliver these adaptations in inactive, but healthy older adults. Studies to date have used two or three training sessions per week but nobody has specifically looked at frequency. Therefore, the aim of this study was to explore the impact of SIT frequency on blood glucose control, aerobic capacity and physical function in older adults. It was hypothesised that twice a week training would produce greater adaptations than training once per week, but both would be effective compared to no exercise. 

## 2. Materials and Methods

### 2.1. Participants

Thirty-four older adults (13 males and 21 females; Age: 65 ± 3 years; Height: 165 ± 8 cm; Body Mass: 73 ± 14 kg) volunteered to take part in the study after local newspaper advertisement and were randomly allocated to the intervention groups first then to the control group. Participants were required to be inactive and were excluded if they had any metabolic disease or cardiovascular disease. All participants had well controlled hypertension (defined as blood pressure ≤160/90 mmHg) with oral hypertensive medication which was unchanged for 6 months. There was no significant difference in baseline characteristics of the three groups (Table 1). Participants allocated to the control group (*n* = 12) were asked to continue with their normal lifestyle throughout the study period. Participants allocated to the SIT groups took part in either a twice- (*n* = 11) or once- (*n* = 11) weekly, 8 week SIT intervention, but no other exercise training. All participants were asked to report any changes in lifestyle or medication during the study. Study information was given verbally and in writing before participants gave verbal and written consent. The study had ethical approval from Abertay University Ethics Committee (SHS0701615/2) and was carried out in line with the declaration of Helsinki.

### 2.2. Baseline Testing

Participants fasted overnight before reporting to the laboratory, where height was recorded using a SECA 217 Stadiometer (SECA United Kingdom, Birmingham, UK) and body mass determined using a SECA Medical 780 weighing scales (SECA United Kingdom, Birmingham, UK). Participants were also asked to refrain from undertaking any strenuous exercise 2 days prior to baseline testing. They then underwent the following tests.

#### 2.2.1. Oral Glucose Tolerance Test

Upon arrival at the laboratory, participants sat for 10 min to ensure that they were fully rested. Following this, a fasting glucose blood sample was taken from the index finger using a single-use lancet (Accu-Chek, Roche Diagnostics Ltd., Sussex, UK) and pressure applied to draw blood from the incision. The initial blood droplet was discarded, with the second blood droplet taken for analysis of blood glucose using a Freestyle Lite Blood Glucose Monitor (Abbott, Chicago, IL, USA). A cotton pad was then placed on the incision with pressure applied. Participants then consumed 75 g of glucose (410 mL Lucozade Original, GlaxoSmithKline, Brentford, UK) as quickly as possible and were encouraged to take no longer than 5 min. Blood glucose concentration was then recorded every 20 min, as described above, for 120 min. 

#### 2.2.2. Get Up and Go Test (GUAG)

Participants started seated, with their arms folded across their chest. They then rose from the chair, without using their arms, and walked 3 m as fast as possible without running, turned 180° and walked 3 m back then sitting down on a chair. This was repeated twice with 2 min recovery between each attempt, with the average time taken reported.

#### 2.2.3. Sit to Stand Test (STS)

Participants started seated with their arms folded across their chest. They rose from the chair, without using their arms and then sat back down on the chair. The total time to carry out five sit to stand repetitions was recorded and the procedure was repeated two times with 2 min recovery between each attempt and the average time taken reported. 

#### 2.2.4. Single-Stage Submaximal Walking Test 

Participants performed a single-stage submaximal walking test as previously described [18] using a Mercury HP Cosmos treadmill (h/p/cosmos sports and medical gmbh, Nussdorf-Traunstein, Germany). Prior to performing the test, participants were fitted with a Polar Heart Rate Monitor (Polar Electro Ltd., Warwick, UK) to allow for heart rate to be monitored throughout the protocol. Following this, participants were required to select a comfortable walking pace and walked against a 0% gradient for four min. The gradient was then increased to 5% and the participants walked for a further 4 min. At the end of this stage heart rate and perceived exertion (Borg Scale) were recorded. An estimation of VO_2_ max was then calculated after the submaximal walking test described previously [18] using the following formula:

VO_2_ max = 15.1 + 21.8 × Speed (mph) − 0.327 × Heart Rate (bpm) − 0.263 × Speed × Age (year) + 0.00504 × Heart Rate × Age + 5.98 × Gender (0 = Female; 1 = Male).

### 2.3. Training Intervention

All SIT sessions took place at Abertay University and were fully supervised. Prior to starting training, a bioharness 2 (Zephyr Technology, Annapolis, MD, USA) was attached to participants to allow continuous monitoring of heart rate (bpm). Participants then performed 6 × 6 second all out cycle sprints (Monark) against 7% body mass for males and 6.5% body mass for females. Resistance was dropped once the participant reached 100 rpm and recovery between sprints was a minimum of 60 s or until heart rate dropped below 120 bpm. Recovery between the sprints was passive to allow heart rate to reduce between sprints. Each group increased sprint number by one each week until the participants were doing 10 × 6 second all out cycle sprints by week 5 of the study. Sprint load was then maintained for the rest of the study and the once a week group completed 70 sprints and the twice a week group completed 140 sprints over 8 weeks.

### 2.4. Post Testing 

All post-tests were repeated in the same order as before, for all groups, after the completion of the intervention phase. There was an average of 5 ± 2 days between the final SIT session and post intervention testing for the SIT groups. 

### 2.5. Data Analysis 

All data are presented as the mean ± standard deviation or the mean ±95% confidence interval. Glucose area under the curve (AUC) was calculated using the conventional trapezoid method. All data were checked for normal distribution using a Shapiro–Wilk test and were within normal values for skewness and kurtosis. A 3 × 2 repeated for time ANOVA was used to determine group × time interaction for all variables. When there was a significant group × time interaction least squares difference post hoc testing was used to determine differences. Significance was accepted at *p* < 0.05 (two-tailed). To infer effectiveness of intervention when there was a significant group × time effect, the absolute change was calculated. Smallest worthwhile change was calculated as 0.2 × standard deviation [19] and 95% confidence intervals calculated. Cohens d was then calculated for absolute change between groups with <0.35 trivial, 0.35–0.8 small, 0.8–1.5 moderate and >1.5 large [20].

## 3. Results

### 3.1. Blood Glucose Control

There was no significant difference between the groups at baseline for any blood glucose variable (Table 1; *p* > 0.05). There was no significant change in fasting glucose in any of the groups (Table 1; *p* > 0.05). There was a significant group × time interaction for 2 h blood glucose (Table 1; *p* = 0.001) and for glucose AUC (Table 1; *p* = 0.027). Following 8 weeks, there was a significant increase in 2 h blood glucose in the control group (Table 1; *p* = 0.023) and a significant decrease in 2 h blood glucose with twice a week SIT (Table 1; *p* = 0.001). There was no significant difference in 2 h blood glucose with once a week SIT (Table 1; *p* = 0.249). Following 8 weeks, there was no significant change in glucose AUC in the control group (Table 1; *p* = 0.324) and a significant decrease in glucose AUC with twice a week SIT (Table 1; *p* = 0.007). There was no significant difference in glucose AUC with once a week SIT (Table 1; *p* = 0.124).

**Figure 1:**
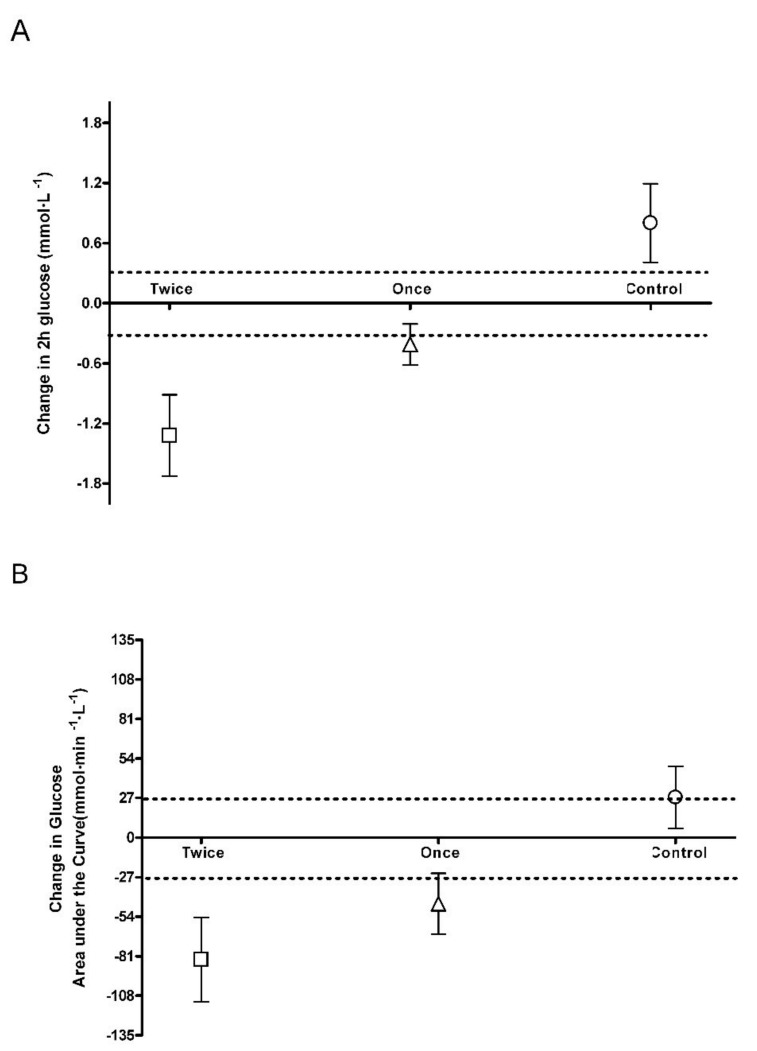
Blood glucose smallest worthwhile change. (**A**): 2 h post blood glucose; (**B**): Glucose area under the curve. Dotted line represents the upper and lower boundary of the smallest worthwhile change.

Following 8 weeks, 2 h blood glucose was decreased in both the once and twice a week group with the average change for each group greater than the smallest worthwhile change (SWC: ±0.3 mmol·L^−1^; Once: −0.41 mmol·L^−1^, 95% CI −0.8, −0.004; Twice: −1.32 mmol·L^−1^, 95% CI −2.1, −0.5; Figure 1A). In the control group there was an increase with the average change greater than the smallest worthwhile change (SWC: ±0.3 mmol·L^−1^; Control: 0.80 mmol·L^−1^, 95% CI 1.5, 0.1; Figure 1A). There was a moderate effect size for the two training groups compared to control for 2 h blood glucose (Control vs. Once: *d* = −1.00; Control vs. Twice: *d* = −1.26) and a small to moderate effect size between training groups (Once vs. Twice: *d* = 0.80). 

Following 8 weeks, glucose AUC was decreased in both the once and twice a week group with the average change for each group greater than the smallest worthwhile change (SWC: ±27.2 mmol·L^−1^·min; Once: −45.2 mmol·L^−1^·min, 95% CI −95.1, 4.8; Twice: −83.3 mmol·L^−1^·min, 95% CI −137, −29.5; Figure 1B). In the control group there was an increase with the average change higher than the smallest worthwhile change (SWC: ±27.2 mmol·L^−1^·min; Control: 27.4 mmol·L^−1^·min, 95% CI 87.4, −33; Figure 1B). There was a small to moderate effect size for the two training groups compared to control for 2h blood glucose (Control vs. Once: *d* = −0.72; Control vs. Twice: *d* = −0.99) and a small effect size between training groups (Once vs. Twice: *d* = 0.43). 

### 3.2. Physical Function

There was no significant difference between the groups at baseline for any physical function variable (Table 1; *p* > 0.05). There was a significant group × time interaction for GUAG (Table 1; *p* = 0.014) and for STS (Table 1; *p* = 0.001). Following 8 weeks, there was no change in the control group for either GUAG (Table 1; *p* = 0.130) or STS (Table 1; *p* = 0.646). Following 8 weeks, there were significant decreases in GUAG (Table 1; Once: *p* = 0.007; Twice: *p* = 0.001) and STS (Table 1; Once: *p* = 0.012; Twice: *p* = 0.040) for both training groups. 

**Figure 2:**
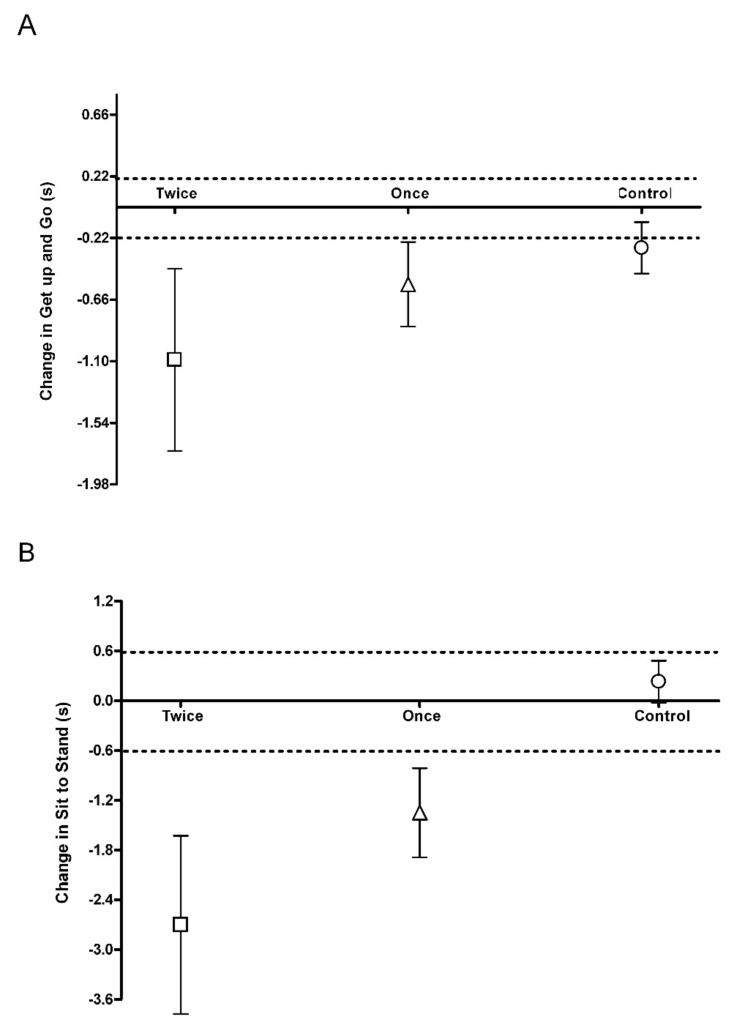
Physical function smallest worthwhile change. (**A**): Get up and go test; (**B**): Sit to stand test. Dotted line represents the upper and lower boundary of the smallest worthwhile change.

Following 8 weeks, GUAG was decreased in both the once and twice a week group with the average change for each group greater than the smallest worthwhile change (SWC: ±0.2 s; Once: −0.55 s, 95% CI −0.8, −0.3; Twice: −1.32 s, 95% CI −1.7, −0.5; Figure 2A). In the control group there was an increase with the average change greater than the smallest worthwhile change (SWC: ±0.2 s; Control: −0.29 s, 95% CI −0.5, −0.1; Figure 2A). There was a small to moderate effect size for the two training groups compared to control for GUAG (Control vs. Once: *d* = −0.69; Control vs. Twice: *d* = −1.00) and a small to moderate effect size between training groups (Once vs. Twice: *d* = 0.69). 

Following 8 weeks, STS was decreased in both the once and twice a week group with the average change for each group greater than the smallest worthwhile change (SWC: ±0.6 s; Once: −1.35 s, 95% CI −2.5, −0.2; Twice: −2.67 s, 95% CI −3.8, −1.6; Figure 2B). In the control group there was an increase with the average change lower than the smallest worthwhile change (SWC: ±0.6 s; Control: 0.22 s, 95% CI 0.8, −0.4; Figure 2B). There was a small to moderate effect size for the two training groups compared to control for STS (Control vs. Once: *d* = −0.72; Control vs. Twice: *d* = −0.99) and a small effect size between training groups (Once vs. Twice: *d* = 0.43).

### 3.3. Predicted VO_2_ Max

There was no significant difference between the groups at baseline for predicted VO_2_ max (Table 1; *p* > 0.05). There was a significant group x time interaction for predicted VO_2_ max (Table 1; *p* = 0.002) with no significant change in the control (Table 1; *p* = 0.257). Following 8 weeks, there was a significant increase in predicted VO_2_ max for both training groups (Table 1; Once: *p* = 0.001; Twice: *p* = 0.001). 

**Figure 3:**
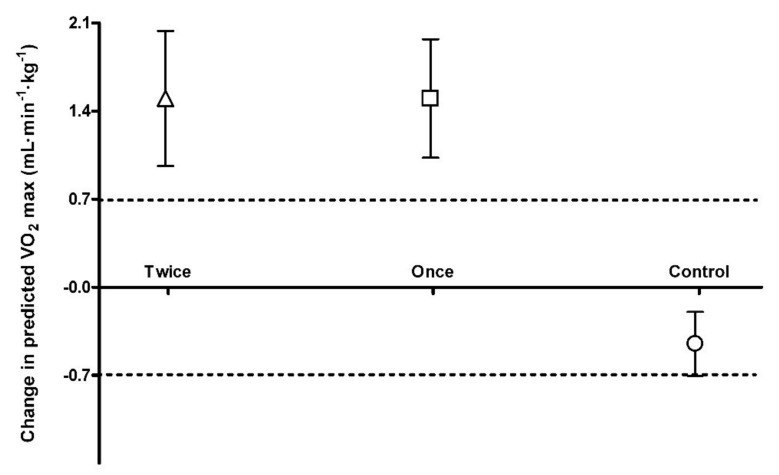
Predicted VO_2_ max smallest worthwhile change. Dotted line represents the upper and lower boundary of the smallest worthwhile change.

Following 8 weeks, predicted VO_2_ max was increased in both the once and twice a week group with the average change for each group greater than the smallest worthwhile change (SWC: ±0.7 mL·min^−1^·kg^−1^; Once: 1.5 mL·min^−1^·kg^−1^, 95% CI 2.3, 0.7; Twice: 1.5 mL·min^−1^·kg^−1^, 95% CI 2.5, 0.5; Figure 3). In the control group there was a decrease with the average change lower than the smallest worthwhile change (SWC: ±0.7 mL·min^−1^·kg^−1^; Control: −0.45 mL·min^−1^·kg^−1^s, 95% CI −0.9, 0.03; Figure 3). There was a moderate effect size for the two training groups compared to control for predicted VO_2_ max (Control vs. Once: *d* = 1.30; Control vs. Twice: *d* = 1.19) and a trivial effect size between training groups (Once vs. Twice: *d* = 0.01).

## 4. Discussion

This is the first study to look at training frequency with sprint interval training in older adults and both training groups produced changes greater than the smallest worthwhile change (Figure 1, Figure 2 and Figure 3). The major finding from this study is that once a week sprint interval training (1 min per week) is sufficient to produce improvements in physical function and aerobic capacity in older adults with only a small or trivial effect found by doubling the training load (Figure 2 and Figure 3). Whilst there was no significant reduction in markers of blood glucose control in the once a week sprint interval training group (Table 1), there was a small to moderate effect compared to control (Figure 1). Doubling the training load produced a small to moderate effect on blood glucose control after 8 weeks of training (Figure 1). This suggests that training once a week using sprint interval training would have a significant impact on the health and wellbeing of older adults but doing it more frequently could have greater metabolic health benefits. This is similar to findings in younger, healthy, inactive adults (Age: 31.7 ± 2.6 years) which suggest 3 times a week high-intensity training produced greater metabolic adaptations than training twice weekly [21]. However, we still need to determine if total sprint numbers were the same, would the differences between the training groups still persist. 

### 4.1. Blood Glucose Control

Following 8 weeks of sprint training, fasting glucose remained unchanged across the groups but there was a significant reduction in 2 h glucose and glucose AUC in the twice a week training group (Table 1). Whereas the once a week training group was not significantly different from baseline but was greater than the smallest worthwhile change (Table 1, Figure 1). However, the effect size between the training groups was only small for glucose AUC and moderate for 2 h blood glucose (Figure 1). It may be that a greater frequency of exercise is required to produce larger adaptations in whole body glucose regulation, or it may just reflect the greater sprint training volume in the twice-weekly group. Potentially, greater frequency of skeletal muscle glycogen turnover with twice-weekly training may lead to greater increases in GLUT4 concentration and glycogen synthase activity [22]. With endurance exercise, low-volume high-intensity training has been shown to have a smaller improvement in glucose metabolism than high volume training [23]. In contrast, 6 months of resistance training had no effect on glucose metabolism regardless of training frequency [24]. The reduction in 2 h glucose and glucose AUC in the twice a week group is similar to that seen with the same sprint protocol performed twice-weekly in middle aged adults [14] and with 30 second sprints performed thrice-weekly in young adults [13]. This suggests similar adaptations in glucose disposal regardless of sprint duration or age. Indeed, when different sprint durations have been compared for performance outcomes in young adults, there has been no difference in adaptation reported [25]. 

### 4.2. Physical Function

Following 8 weeks of sprint training, there was a significant reduction in timed get up and go and sit to stand in both once and twice a week training groups (Table 1). Both groups produced improvements in physical function that was greater than the smallest worthwhile change (Figure 2) and the once a week group had a small effect compared to control and the twice a week group a moderate effect compared to the control, which is similar to that reported for thrice-weekly strength training [26]. However, pooled data on timed get up and go show no effect of strength training on this physical function test [27]. This may suggest that once- or twice-weekly SIT may be a more effective way to improve wider aspects of physical function in older adults compared to strength training. The size of improvement of 16% in the twice-weekly training group was similar, whereas the improvement of 7% in the once-weekly group was smaller than previously reported 11% [15]. There was a small to moderate effect size between the training groups for both physical function tests (Figure 2), which suggests an additional benefit by doubling the training frequency. This may be due to a greater neuromuscular adaptation with the greater frequency. With 2 min of additional twice-weekly high-intensity sprint training there is a greater motor unit activation compared to only endurance training [28]. However, we cannot rule out that this is a training volume effect rather than frequency effect. 

### 4.3. Predicted VO_2_ max

Following 8 weeks of sprint training, there was a significant increase in predicted VO_2_ max in both once and twice a week training groups (Table 1). The improvement was bigger than the smallest worthwhile change in both groups (Figure 3). This size of improvement (5%) in aerobic capacity is similar for both training groups and to previous studies in different populations [15,24]. Greater aerobic capacity in older adults has been associated with greater physical function and may, to some extent, explain the improved performance in functional tests [29]. The effect size between the training groups was trivial for the predicted VO_2_ max (Figure 3). This suggests that adaptation of aerobic metabolism is an early response to sprint training and that increasing frequency of training has no additional benefits. Such findings are in agreement with a recent study by Stavrinou et al. [21], who reported that different high-intensity interval training frequency (two vs. three times per week) similarly affects the magnitude of improvement in VO_2_ peak in young, inactive, healthy adults. Furthermore, it has been shown that in young adults, changes in VO_2_ max occur within six sprint sessions and then plateau regardless of training volume [25].

## 5. Conclusions

In conclusion, we demonstrate for the first time the impact of training frequency on adaptations to sprint interval training in older adults. Training frequency is known to be important for improvements in a number of different aspects of fitness and health [26]. We demonstrate that twice- and even once-weekly sprint interval training significantly improves both physical function and aerobic fitness in older adults but only twice-weekly training improves whole body glucose metabolism. The fact that only once a week is sufficient to produce health benefits means that the minimum time and frequency of exercise required is much lower than currently recommended [30]. These are important findings given the number of older adults who fail to reach the recommended physical activity levels [31] and the projection for population demographics across countries [32]. Therefore, our results provide further support for the inclusion of high-intensity interval training in the latest physical activity guidelines as one of the methods to gain health benefits by accumulating vigorous intensity physical activity. However, further research is needed to explore the impact of frequency when total sprint volume is the same across training groups.

## Figures and Tables

**Table 1:** Participant characteristics and response to training. (a = group × time effect *p* < 0.02; b = *p* < 0.01 pre vs. post; c = *p* < 0.05 pre vs. post).

Characteristic	Control (*n* = 12; 2 M, 10 F)		Once (*n* = 11; 4 M, 7 F)		Twice (*n* = 11; 5 M, 6 F)	
	Pre	Post	Pre	Post	Pre	Post
Age (year)	65 ± 3	65 ± 3	65 ± 4	65 ± 4	66 ± 4	66 ± 4
Height (cm)	162 ± 6	162 ± 6	165 ± 7	165 ± 7	169 ± 10	169 ± 10
Weight (kg)	68 ± 11	69 ± 12	74 ± 14	74 ± 15	77 ± 17	77 ± 17
Body Mass Index (kg·m^−2^)	26.0 ± 4.3	26.2 ± 4.4	27.1 ± 4.2	27.0 ± 4.1	26.8 ± 4.1	26.7 ± 4.1
Predicted VO_2_ max (mL·min^−1^·kg^−1^)	30.0 ± 1.8	29.6 ± 2.0 ^a^	29.0 ± 4.5	30.5 ± 5.2 ^a,b^	29.7 ± 4.2	31.2 ± 3.8 ^a,b^
Blood Glucose						
Fasting (mmol·L^−1^)	5.4 ± 0.4	5.2 ± 0.8	5.3 ± 0.4	5.1 ± 0.1	5.6 ± 0.8	5.2 ± 0.9
2 h (mmol·L^−1^)	7.1 ± 0.9	7.9 ± 1.2 ^a,c^	7.4 ± 1.0	7.0 ± 0.7 ^a^	8.1 ± 1.7	6.8 ± 1.8 ^a,b^
Area under curve (mmol·min·L^−1^)	975 ± 96	1003 ± 101 ^a^	1027 ± 113	981 ± 126 ^a^	1058 ± 184	975 ± 183 ^a,b^
Physical Function						
Get up and Go (s)	7.0 ± 1.1	6.7 ± 1.1 ^a^	6.7 ± 0.9	6.2 ± 0.7 ^a,b^	7.0 ± 1.2	5.9 ± 0.5 ^a,b^
Sit to Stand (s)	12.1 ± 4.3	12.3 ± 4.2 ^a^	11.9 ± 1.8	10.6 ± 2.1 ^a,b^	12.0 ± 2.1	9.3 ± 1.1 ^a,c^
